# 

**Published:** 1897-03

**Authors:** 


### COMMITTEES FOR TRI-UNION MEETING OF THE WASHINGTON CITY DENTAL SOCIETY, MARYLAND STATE DENTAL ASSOCIATION AND THE VIRGINIA STATE DENTAL ASSOCIATION, 1897.

The Tri-Union Meeting of the Washington City Dental Society and the Maryland State and Virginia State Dental Associations will be held Thursday, Friday and Saturday, May 6, 7, and 8, 1897, at Old Point Comfort, Va.

Previous Union meetings have been largely attended, great satisfaction has been expresse l with their outcome, and papers of rare value have been presented and discussed.

A special feature of this meeting will be the presence of a large number of clinicians, who will practically demonstrate the latest theories of advanced dentistry. Dr. B. Holly Smith, of Baltimore, Md., will have personal control of this part of the program.

Every member is expected to contribute something, even if it be but an "incident of office practice." Every contributor, clinician and chairman of committee should report by April 10, 1897, to Dr. D. N. Rust, Secretary Joint Committee, 122 S. Washington St., Alexandria, Va.

R. G.

### COMMITTEES.

Publication and Essays: W. W. Dunbracco, Chairman, 1023 Edmondson avenue, Baltimore, Md.; G. M. Smith, Baltimore, Md.; W. W. H. Thackston, Farmville, Va.; C. C. Harris, Baltimore, Md.; Wm. Hunt, Washington. D. C.; W. E. Norris, Charlottesville, Va.; W. N. Cogan, Washington, D. C.

Anatomy, Physiology and Histology: D. E. Wiber, Chairman, 1210 F Street, N. W., Washington, D. C.; W. A. Lyon, Washington, D. C.; J. A. Colvin, Charlottesville, Va.; W. M. Ash, Washington, D. C.; Edward Nelson, Frederick, Md.; W. D. Wood, Suffolk, Va.; A. J. Volck, Baltimore, Md.

Operative Dentistry: Chas. L. Steel, Chairman, 723 Main street, Richmond, Va.; D. N. Rust, Alexandria, Va.; T. H. Davy, Baltimore, Md.; R. H. Walker, Norfolk, Va.; A. D. Cohey, Washington, D. C.; J. E. Orrison, Baltimore, Md.; A. J. Brown, Washington, D. C.

Prosthetic Dentistry: W. B. Finney, Chairman, 813 N. Eutaw street, Baltimore, Md.; J. W. Smith, Baltimore, Md.; J. O. Hodgkin, Warrenton, Va.; C. J. Grieves, Baltimore, Md.; J, H. Lewis, Washington, D. C.; Edw. Eggleston, Richmond, Va.; J. R. Walton, Washington, D. C.

Dental Education, Literature ahd Nomenclature: H. C. Thompson, Chairman, 1113 Penna. avenue, Washington, D. C; Geo. B. Welch, Washington, D. C.; J. W. Woodley, Norfolk, Va.; W. S. Harban, Washington, D. C.; W. A. Montell, Baltimore, Md.; Geo. F. Keesel, Richmond, Va.; Richard Grady, Baltimore, Md.

Pathology and Therapeutics: H. W. Campbell, Chairman. Suffolk, Va.; W. H. Ewald, Wytheville, Va.; G. H. Claude, Annapolis, Md.; E. P. Beadles, Danville, Va.; W. E. Dieffenderfer, Washington, D. C.; W. A. Mills, Baltimore, Md.: J. R. Hagan, Washington, D. C.

Crown and Bridge-work: Geo. E. Hardey, Chairman, 716 Park avenue, Baltimore, Md.; Jos. Roach, Balmore, Md.; S. J. Cockerille, Jr., Washington, D. C.; E. E. Cruzen, Cumberland, Md.; J. L. Walker, ———, Va.; S. G. Davls, Washington, D. C.; Geo. K. Heist, Winchester, Va.

Orthodontia and Dental Appliances: H. B. Noble, Chairman, 1324 New York avenue, Washington, D. C.; C. W. Appler, Washington, D. C.; R. T. McQuown, Abingdon, Va.; H. M. Schooley, Washington, D. C.; M. G. Sykes, Ellicott City, Md.; A. R. Bowles, Richmond, Va.; David Genese, Baltimore, Md.

Oral Surgery: Jas. F. Thompson. Chairman, Fredericksburg, Va.; W. S. Gregory, Roanoke, Va.; A. W. Lakin, Boonsboro, Md.; T. H. Parramore, Hampton, Va.; H. J. Allen, Washington, D. C., J. H. Harris, Baltimore, Md.; L. C. F. Hugo, Washington, D. C.

Anæsthetics: F. J. S. Gorgas, Chairman, 845 N. Eutaw Street, Baltimore, Md.; Samuel C. Pennington, Baltimore, Md.; J. C. Smythe, Washington, D. C.; Philip Sasscer, Waldorf, Md.; A. H. Sprinkel, Culpepper, Va.; R. W. Talbott, Washington, D. C.; F. A. Lee, Lynchburg, Va.

Hygiene: M. F. Finley, Chairman, 1928 I Street, N. W., Washington, D. C.; J. L. Wolf, Washington, D. C.; L. M. Cowardin, Richmond, Va.; F. M. Seibold, Washington, D. C.;

M. W. Foster, Baltimore, Md.; C. A Mercer, Richmond, Va.; A. P. Gore, Baltimore, Md.

Dental Legislation: B. Bridgforth, Chairman, Richmond, Va.; W. W. Moss, Charlottesville, Va.; A. C. McCurdy, Towson, Md.; Wm. Farmer Wytheville, Va.; R. H. Gunnell, Washington, D. C.; E. P. Keech, Baltimore, Md:; E. K. Gerow, Washington, D. C.

---

### Monthly Summary.

### To Readers and Correspondents.

Communications intended for publication, and exchange journals and papers, should be addressed to the Editor of The American Journal of Dental Science, No. 845 N. Eutaw St. Baltimore, Md. All letters relating to the business of the Journal, or containing cash remittances, to be directed to Snowden & Cowman Mfg. Co., 9 W. Fayette Street, Baltimore, Md. Articles for publication should be received by the Editor at least two weeks previous to the first of the month on which the number in which it is designed for them to appear is issued.

☞ The American Journal of Dental Science will contain fifty pages of reading matter in each number, making a volume of about Six Hundred pages,

EXCLUSIVE OF ADVERTISEMENTS.

